# Smart Surgical Catheter for C‐Reactive Protein Sensing Based on an Imperceptible Organic Transistor

**DOI:** 10.1002/advs.201701053

**Published:** 2018-05-02

**Authors:** Xudong Ji, Pengcheng Zhou, Ling Zhong, Aimin Xu, Anderson C. O. Tsang, Paddy K. L. Chan

**Affiliations:** ^1^ Department of Mechanical Engineering The University of Hong Kong Hong Kong; ^2^ State Key Laboratory of Pharmaceutical Biotechnology The University of Hong Kong Hong Kong; ^3^ Department of Medicine The University of Hong Kong Hong Kong; ^4^ China Department of Pharmacology and Pharmacy The University of Hong Kong Hong Kong; ^5^ Department of Surgery The University of Hong Kong Hong Kong

**Keywords:** bending stability, biomedical applications, C‐reactive protein (CRP) sensors, organic transistors, ultraflexibility

## Abstract

Organic field‐effect transistors (OFETs)‐based sensors have a great potential to be integrated with the next generation smart surgical tools for monitoring different real‐time signals during surgery. However, allowing ultraflexible OFETs to have compatibility with standard medical sterilization procedures remains challenging. A novel capsule‐like OFET structure is demonstrated by utilizing the fluoropolymer CYTOP to serve both encapsulation and peeling‐off enhancement purposes. By adapting a thermally stable organic semiconductor, 2,10‐diphenylbis[1]benzothieno[2,3‐d;2′,3′‐d′]naphtho[2,3‐b;6,7‐b′]dithiophene (DPh‐BBTNDT), these devices show excellent stability in their electrical performance after sterilizing under boiling water and 100 °C‐saturated steam for 30 min. The ultrathin thickness (630 nm) enables the device to have superb mechanical flexibility with smallest bending radius down to 1.5 µm, which is essential for application on the highly tortuous medical catheter inside the human body. By immobilizing anti‐human C‐reactive protein (CRP) (an inflammation biomarker) monoclonal antibody on an extended gate of the OFET, a sensitivity for detecting CRP antigen down to 1 µg mL^−1^ can be achieved. An ecofriendly water floatation method realized by employing the wettability difference between CYTOP and polyacrylonitrile (PAN) can be used to transfer the device on a ventricular catheter, which successfully distinguishes an inflammatory patient from a healthy one.

The development of organic field‐effect transistors (OFETs)‐based sensors has attracted increasing attention recently for their biomedical and conformal applications on organ surface^1^, ^2^ or human skin.^3^, ^4^ Various physical signal sensors such as pulse,^5^ temperature,^6^ skin pressure,^7^ blood pressure,^8^ or electrical signal sensors like ECG,^9^ EEG,^10^ EMG^11^ have been successfully demonstrated. These devices can provide real‐time information of human body or particular organs. Besides these physical and electrical signals, surgical tools integrated with OFET‐based sensors to measure the chemical and biological signals are also critical for monitoring the health conditions of patients during surgery. For example, C‐reactive protein (CRP), a biomarker of inflammation widely distributed in our blood stream, can be used to indicate the levels of inflammation in our body. A normal CRP value in human blood is usually below than 10 µg mL^−1^, while in inflammation case, the CRP level can go up to 40 µg mL^−1^. Under the bacterial inflammation, the CRP concentration in our blood can be as high as 100 µg mL^−1^.^12^ As a result, if a surgical tool like catheter can be integrated with a CRP sensor to monitor the real‐time inflammation level during cardiac surgery, especially cardiopulmonary bypass (CPB), it would be more than useful for doctors to promptly treat systemic inflammation, which is a primary cause of various postoperative complications leading to vital organ dysfunction and multiorgan failure.^13^ To allow the application of these new sensors in the operating room, it is essential for the OFETs to withstand the standard thermal sterilization procedure while at the same time they need to be ultraflexible and conformal for integrating onto medical tools. Traditionally, this is the weak side of the organic semiconductors due to their relativity low thermal stability^14^, ^15^ and high sensitivity to the moisture or oxygen in the air which needs to be addressed. As the standard clinical sterilization processes are performed under boiling water or saturated steam with a temperature of 100 °C,^16^, ^17^ it is necessary to use a thermally stable semiconductor as well as a robust encapsulation in order to make the device survive under such harsh conditions. Recently, Takimiya et al. reported a family of thienoacene‐based organic semiconductors with very good thermal stability.^18^ Based on the dinaphtho[2,3‐*b*:2′,3′‐*f*]thieno[3,2‐*b*]thiophene (DNTT), Kuribara et al. adapted the gold/parylene hybrid encapsulation layer to develop the first organic transistor with the sterilization compatibility in 2012.^16^ However, the relativity thick polyimide substrate about 75 µm would significantly limit the flexibility of the whole device and their adaptability onto different surgical tools such as aneurism clips, catheters or tweezers. Very recently, Kyaw et al. reported a polymer transistor with thermal stability up to 350 °C under nitrogen environment. By using a very thick (115 µm) polypropylene and polychlorotrifluoroethylene encapsulation layer, these devices are suitable for the autoclave sterilization^19^ with a temperature up to 121 °C for 30 min, while their flexibility and conformability are severely restricted. As a result, there is a strong desire to develop a universal OFET structure with not only high sterilization compatibility but also high mechanical flexibility, which can be integrated onto different surgical tools for clinical applications.

To achieve high mechanical flexibility, the overall device thickness including active layer, substrate, and encapsulation layer down to a few micrometers or sub‐micrometer level is essential. To date, these ultrathin OFETs are supported by a rigid frame during fabrication. Then they will be delaminated and transferred onto the target surface for applications. Mechanical peeling or chemical etching is the two commonly used delamination methods. In the mechanical method, the ultrathin devices are generally peeled off from the polydimethylsiloxane (PDMS) stamps or parylene release layer by mechanical forces.^8^, ^20^, ^21^, ^22^, ^23^, ^24^ While in the chemical method, the devices are released by dissolving different sacrificial layers like polyvinyl alcohol (PVA)^25^, ^26^ or polymethyl methacrylate (PMMA)^27^, ^28^, ^29^ with the corresponding solvents. To avoid the fragile properties of the ultrathin substrate during mechanical peeling and the potential hazards to the devices during chemical etching, an easy, integrated and ecofriendly device transfer method need to be used. CYTOP, an amorphous fluoropolymer with high chemical resistance and low gas permeability, is considered as a decent candidate for robust encapsulation.^30^, ^31^, ^32^ Since it is a very hydrophobic polymer with a water contact angle over 110°, we can use it to integrate with a hydrophilic polymer like polyacrylonitrile (PAN) to form a hybrid substrate which would be easily detached under water environment.^33^, ^34^

Here, we develop our OFET under a capsule‐like structure with CYTOP serving as both encapsulation and peeling‐off promoting layer. The overall thickness of the device is only 630 nm and it can withstand boiling water or saturated steam sterilization. After exposing to these extreme conditions for 30 min, the threshold voltage shows only 0.21 V shift while the carrier mobility decreased by only 6.6%. Under the utmost bending radius down to 1.5 µm, the carrier mobility shows negligible change after 5000 bending cycles. By integrating such OFET with extended CRP sensing gate, we can achieve a CRP sensitivity down to 1 µg mL^−1^. This OFET‐based sensor can be transferred onto a ventricular catheter to distinguish between the blood serum from the healthy patient and the mild inflammatory patient. Our devices and findings can accelerate the development of smart surgical tools for real‐time sensing of biomarkers during surgery.

We start with reporting the structure of our flexible organic transistors fabricated on 250 nm PAN/CYTOP hybrid substrate as shown in **Figure** **1**a. The detailed fabrication procedures are shown in the experimental section and illustrated in Figure S1 (Supporting Information). We formed the ultrathin PAN/CYTOP hybrid substrate by two‐time spin coating. The atomic force microscope (AFM) images in Figure S2 (Supporting Information) show a root mean square (RMS) roughness of 0.7 nm with a total film thickness of 250 nm (180 nm PAN and 70 nm CYTOP). To maintain an operating voltage lower than 3 V for portable applications, the high‐k alumina dielectric insulator was deposited by anodization process and treated with octadecylphosphonic acid (ODPA) self‐assembled monolayer (SAM). The active layer organic semiconductor we used is 2,10‐diphenylbis[1]benzothieno[2,3‐d;2′,3′‐d′]naphtho[2,3‐ b;6,7‐b′]dithiophene (DPh‐BBTNDT) which has excellent thermal stability.^35^ The thermal stability of DPh‐BBTNDT was confirmed by the control sample developed on the octadecyltrichlorosilane (OTS)‐treated Si/SiO_2_ substrate (Figure S4, Supporting Information). A top CYTOP layer with 240 nm thickness was used to encapsulate the whole OFETs after the deposition of the top source–drain electrodes. The output and transfer curves of the ultrathin device are shown in Figure 1b,c, respectively. The average carrier mobility over thirty devices is 4.16 cm^2^ V^−1^ s^−1^ with a standard deviation 0.73 cm^2^ V^−1^ s^−1^. The average on‐off ratio is about 10^7^, the threshold voltage (*V*_th_) is −1.64 V (Figure 1d). All the devices have a leakage current less than 100 pA under a field of 3 MV cm^−1^. It is important to notice that the electrical performance of device on the ultrathin substrate with high‐k dielectric is highly comparable with the one fabricated on the Si/SiO_2_ substrate. In the group of thienoacene‐based organic semiconductors developed on high‐k dielectric, our current mobility is one of the highest among the reported values.^36^, ^37^, ^38^ It confirms the fabrication recipe of our alumina dielectric, SAM and active layer are very suitable for the ultraflexible OFETs fabrication.^4^, ^39^

**Figure 1:**
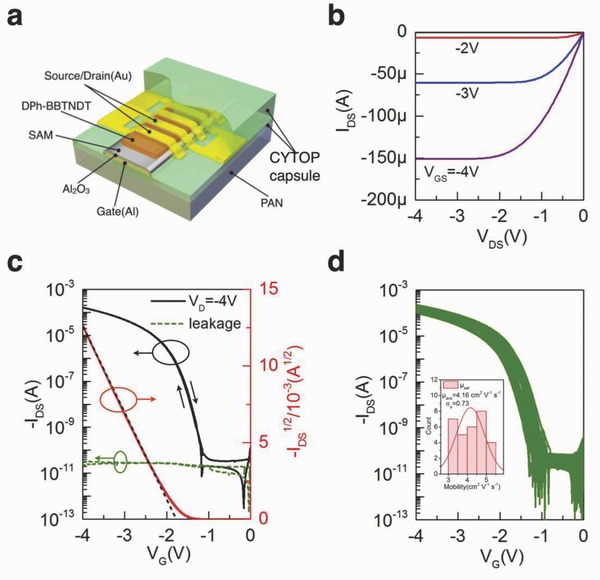
a) OFET structure based on high‐k alumina/ODPA SAM dielectric and ultrathin PAN/CYTOP hybrid substrate (*W*/*L* = 35). b) Output curves at different gate biases (−2 to −4 V with 1 V step). c) Bidirectional sweep of transfer curves (black), square root of drain source current (red) and leakage current (green) at *V*_D_ = −4 V. d) Transfer curves of 30 OFET devices and corresponding mobility histogram, average mobility is 4.16 cm^2^ V^−1^ s^−1^ with standard deviation 0.73 cm^2^ V^−1^ s^−1^.

The device performance before and after 100 °C‐saturated steam sterilization for 30 min is shown in **Figure** **2**a and Movie S1 (Supporting Information). Only slightly change (0.1 V) in the threshold voltage and decrease in carrier mobility (4.6%) can be observed (Figure 2b,c). The robustness of the anodized alumina with ODPA can be reflected by the low leakage current after sterilizing. Other than the hot steam, we also put the device into the boiling water (Figure 2d and Movie S2, Supporting Information) for sterilization directly. A similar trend of negatively shift of threshold voltage (0.21 V) and slightly decrease of mobility (6.4%) were observed, while the leakage current level remained lower than 100 pA (Figure 2e,f). In order to achieve such outstanding sterilization compatibility under high temperature and moisture environment, other than the dielectric and thermally stable DPh‐BBTNDT, the capsule‐like CYTOP encapsulation particularly plays a critical role. The CYTOP capsule can block the vapor and liquid water from diffusing into the device. Even the water vapor transmission rate (WVTR) of CYTOP is only 0.1 g m^−2^ day^−1^,^40^ the hydrophobic nature of CYTOP can prevent the water vapor adsorption before permeation and it is already enough for the 30 min sterilization process.

**Figure 2:**
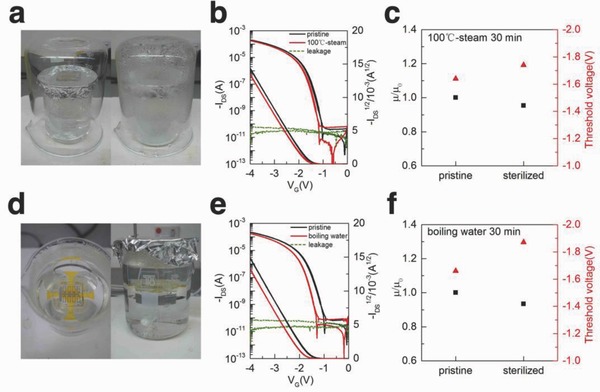
a,d) Photograph of OFET device before and during 100 °C‐saturated steam or boiling water sterilization. b,e) Transfer curves, square root of drain source current before and after 30 min' sterilization and leakage current after 30 min' sterilization. c,f) Normalized mobility and threshold voltage change after 30 min' sterilization.

One major advantage of ultraflexible device is the transferability to different kinds of surface, including medical devices, and allow it to follow the surface morphology without downgrading the functions. The usage of a hydrophobic/hydrophilic double‐ layer substrate structure can facilitate the transfer process from a temporary holder to various surfaces (Figure S6, Supporting Information). To demonstrate the consecutive transferability, we continually transferred eight devices in the same PAN/CYTOP substrate from pristine glass to another glass, polyethylene–naphthalate (PEN) and a Hong Kong banknote (inset images in **Figure** **3**b). The representative transfer curves and leakage current after each transferring process are shown in Figure 3a. Around 0.26 V threshold voltage shift was observed after the first transfer and threshold voltage shift in followed transfer processes are negligible. The shift of *V*_th_ in the first transfer is attributed to the relaxation of the intrinsic strains in the metal contacts and dielectric/semiconductor interface. Other than the shifting of the threshold voltage, the device basically can maintain high on–off ratio and low leakage with negligible variation among different transfer stages. Figure 3b shows the average mobility of all the devices after each transfer process and normalized by the pristine mobility on the glass substrate. Both the carrier mobility and the area capacitance of the dielectric insulator have ignorable change. The area capacitance maintained stable value around 370 nF cm^−2^ during different transfer stages.

**Figure 3:**
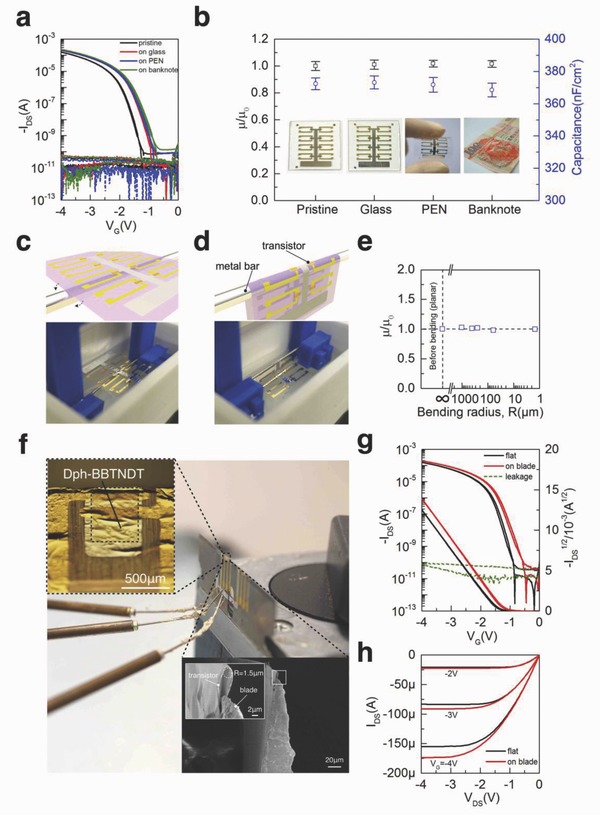
a) Transfer curves and leakage current of OFET devices before and after continuously transferred to a rigid glass, flexible PEN and rough banknote. b) Normalized mobility change and area capacitance of gate dielectric change during each transfer stages. Scale bar is obtained from 8 individual devices. c) Flat state of ultra‐thin OFET device (float on water surface). d) Bending state of ultrathin OFET device lifted by a metal bar with different diameter (2 mm, 800, 500, 120 µm) or a blade with tip diameter 3 µm. e) Normalized mobility change against different bending radius with 5000 bending cycles. f) Photograph of the OFET on blade tip; Optical microscope image of a single OFET on a blade tip shown the active channel of OFET is placed across the tip; Scanning electron microscope (SEM) image shown the device is under intensive bending with bending radius 1.5 µm. g,h) Transfer curves and output curves of the device before and after transferred on blade as well as leakage current of the device on blade.

Apart from demonstrating the transferability, we also utilized a tailor‐made bending test machine as shown in Figure S7 (Supporting Information) to verify the flexibility of these ultrathin devices (Movie S3, Supporting Information). In the bending tests, metal bars with different diameters ranging between 2 mm to 120 µm (Figure 3c,d) and a sharp blade (tip diameter 3 µm, inset of Figure 3f) were used to lift the devices up and down continuously. We applied 5000 cycles bending of all different radii and tested the transfer curves of the devices. All the devices showed negligible change in the mobility, slightly positive shift of *V*_th_ and low leakage current (below 100 pA) after 5000 bending cycles. (Figure 3e and Figure S8, Supporting Information). These findings suggest the degradation of the OFET performance due to bending is actually very limited other than the *V*_th_ shift cause by the substrate transfer. This bending stability can be further confirmed by performing in situ transfer and output measurements while the device is under extreme bending (on blade, Figure 3f). From the inset optical image in Figure 3f, we can notice that the device channel is perpendicular to the bending direction. The device shows negligible change in mobility (3.8%) and *V*_th_ shifts around 0.24 V while it is under extreme bending condition (1.5 µm bending radius) (Figure 3g,h). As shown earlier, we believe that this threshold voltage shift is just induced by the substrate transfer process rather than the bending strain. We evaluated this strain in each layer by the standard mechanical stress–strain analysis^24^ (Figure S9, Supporting Information). When the bending radius was 1.5 µm, a 0.56 % tensile strain is present between the dielectric and semiconductor interface, which is smaller than the reported strain value (≈2%) where irreversible degradation starts to occur.^41^, ^42^ To the best on our knowledge, the bending radius of 1.5 µm is the smallest reported value for the OFETs with flexible substrate down to submicron thickness.^24^, ^34^ We believe that the high quality anodized growth alumina on the ultrathin PAN/CYTOP hybrid substrate is the key leading to such high performance. The alumina dielectric with large Young's modulus (300 GPa) can bring the normal strain plane toward the dielectric and semiconductor interface, which can significantly suppress the stains resulted in the semiconductor layer. While for other devices with smaller Young's modulus dielectric, the normal strain plane will be brought away from the dielectric and semiconductor interface caused by the high Young's modulus back metal gate. This will result in a strong tensile strain in organic active layer especially under a small bending radius. The capability of withstanding small bending radius demonstrates the potential applications of these ultrathin devices on irregular surfaces such as human skin or surgical tools.

To convert the transistor into a CRP sensor, we employed an extended gate structure functionalized with CRP antibody (**Figure** **4**a). The extended‐gate OFETs have been widely used for biological sensing.^43^, ^44^, ^45^, ^46^ However, these devices are usually on the rigid substrates like silicone, glass, or relatively think flexible PEN substrate, which limit their compatibility in the surgical tools, especially those with curved or tortuous shape. In our devices, the aluminum gate was connected with the gold extended gate, which was functionalized with CRP antibody and blocked with bovine serum albumin (BSA) protein to avoid nonspecific binding. The detailed functionalization steps are described in Experimental Section. In the CRP sensor configuration, the Ag/AgCl gate and the functionalized Au electrode are connected in series through phosphate buffered saline (PBS) solution and thus the functionalized Au electrode can be considered as a floating gate electrode, which can effectively modulate the net gate bias onto the device by regulating its surface potential during antibody–antigen combination. Debye's screening length is deemed to be an important parameter in electrochemical detection of biomolecules. It is believed that for the traditional bottom gate FETs where proteins are directly attached onto the channel area, the sensitivity of these devices are largely limited by the Debye's screening length.^47^ However, for electrolyte‐gated transistor or extended gate transistor in this work, the sensing effect are attributed to the modification of the overall capacitance^48^ as well as threshold voltage shift induced by the surface potential on the extended gate.^49^, ^50^ Therefore, the Debye's screening length, which is expected to dominate in electrostatic detection, would not affect a lot in these kinds of sensors. We tested the sensors by adding in CRP antigen with different concentration ranging from 100 ng mL^−1^ to 10 µg mL^−1^ into the PDMS reservoir and monitored the channel current change of the transistor (Figure 4b). The channel current started to increase after 1 µg mL^−1^ CRP antigen was introduced, and then the current elevated with the CRP antigen concentration. The sensitivity limit around 1 µg mL^−1^ is sufficient to sense the normal CRP antigen concentration in human blood and the change of the current signal in the sensor shows linear response in the semi‐log plot against CRP antigen concentration (Figure 4c). A comparison table of CRP sensors with different structures can be found in Table S2 of the Supporting Information. After obtained the calibration plot, we transferred the device to a ventricular catheter to mimic the CRP antigen sensing inside the blood vessel. An illustrative image is shown in Figure 4d and the exploded view of the device is shown in Figure S11 (Supporting Information). The electrical connection on such catheter was obtained by thermally evaporated silver on PEN substrate which were fixed on the catheter with 2 mm diameter. The ultrathin transistor was transferred to the catheter and followed by transferring of a separated fabricated extended gate. The interconnector, transistor, and extended gate were connected by vertical interconnect access (VIA) connection. As shown in the solid lines in Figure 4e, the sensor shows sharp response in the blood serum with 20 µg mL^−1^ CRP antigen (inflammatory case), while the response in the blood serum with 2 µg mL^−1^ CRP antigen is much smaller. In the controlled devices without any CRP antibody but only BSA on the extended gate, the current responses are ignorable. It confirms that the current increase in our sensors is contributed to the specific binding between the CRP antigen and antibody on the extended gate.

**Figure 4:**
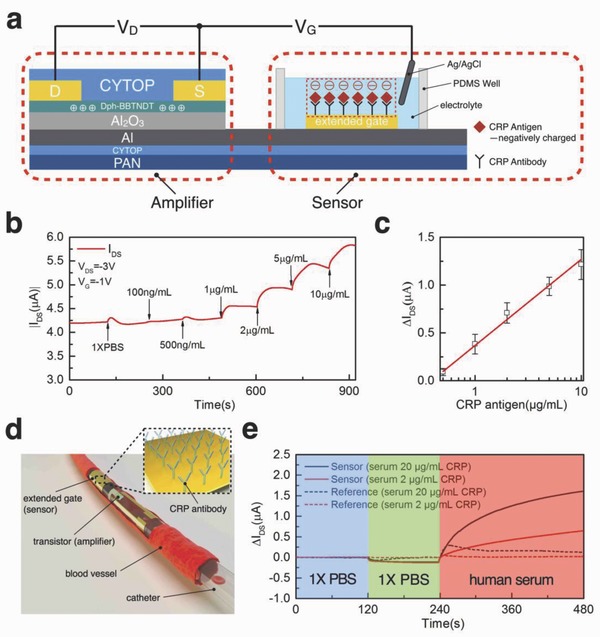
a) Structure of OFET‐based CRP sensor and the sensing mechanism. b) Channel current increase against CRP concentration, *V*_DS_ was fixed at −3 V and *V*_G_ was fixed at −1 V. c) Calibration curve of channel current increase against CRP concentration, error bar was established among five devices. d) Conceptual image of a CRP sensor in ventricular catheter implanted in blood vessel. e) Response of sensor and reference devices on human serum with different concentration of CRP antigen.

In summary, by a combination of a thermally stable organic semiconductor and an ultrathin capsule‐like CYTOP structure, we have successfully fabricated an imperceptible organic transistor with thickness as thin as 630 nm. Such device shows not only high compatibility with medical sterilization (boiling water or 100 °C‐saturated steam) but also extreme bending stability with bending radius as small as 1.5 µm. By using the hydrophobic/hydrophilic hybrid substrate, the imperceptible device can be continuously transferred between various kinds of surfaces with conformable attachment. We further utilize this advantage and fabricated a CRP sensor on ventricular catheter to distinguish different inflammation states of the patients. The proposed capsule‐like device structure having excellent flexibility and compatibility with the medical sterilization process can be an important cornerstone connecting different OFET‐based sensors to the clinical application which can potentially minimize the risk of the patients during surgery.

## Experimental Section

*Fabrication of OFET Device*: First, 180 nm PAN was spin‐coated on precleaned glass substrate from its DMF solution (30 mg mL^−1^) followed by baking at 90 °C for 30 min. Then 2.25 wt% CYTOP solution was spin‐coated on PAN thin film with a thickness 70 nm followed by 100 °C baking for another 30 min. 50 nm aluminum (Al) gate electrode was deposited onto PAN/CYTOP hybrid substrate (250 nm) by thermal evaporation through a shadow mask. A 10 nm thick alumina layer was formed through anodization process with a current density 0.7 mA cm^−2^.^39^ The substrate was then immersed in a 2‐propanol solution of ODPA (2 × 10^−3^ m) to form a SAM on the surface of the oxidized gate. A 30 nm thick film of DPh‐BBTNDT was then deposited through a shadow mask by thermal evaporation with substrate temperature 100 °C, followed by the deposition of 50 nm thick gold through another shadow mask to define the source/drain contacts. Finally, 4.5 wt% CYTOP solution was spin‐coated to form a 240 nm encapsulation followed by 100 °C baking for 1 h.

*Functionalization of Extended Gate*: A gold electrode was deposited on 250 nm PAN/CYTOP substrate by thermal evaporation (Cr/Au, 5 nm/50 nm) serving as extended gate. The electrode was rinsed by deionized water, acetone, and ethanol and followed by 15 min' UV‐Ozone treatment. After that, it was immersed in an ethanol solution of MPA (15 × 10^−3^ m) in glove box for 16 h to form a SAM with carboxylic acid (—COOH) group. Then, the terminal carboxylic acid groups were activated in a solution of NHS/EDC (0.1 m/0.1 m) for 1 h at room temperature. After rinsing with DI water, the extended gate electrode was incubated for 4 h in a 10 µg mL^−1^ solution of CRP antibody. The terminal amine groups on the antibody enable covalent bonding to occur through the activated carboxylic acid group from MPA. Finally, after rinsing with phosphate buffered saline (PBS), the extended gate electrode was incubated for 1 h in 1% BSA solution, which blocks the remaining carboxylic acid groups.

*Transfer of Device on Catheter*: Tiny holes were punched on the contact pad of transistor and extended gate electrode by a needle for VIA connection, then the edge of the substrate is gently scratched to realize the water floatation of device. A tweezers was used to clamp the edge of transistor with a titled angle followed by carefully immersing in a beaker containing water to float the ultrathin transistor. The extended gate was peeled off using same manner. After that, a ventricular catheter with fixed interconnector was used to lift up the ultrathin freestanding device with contact pad laminated. Then the catheter was placed in a desiccator for 1 h to evaporate the water. Silver paste was smeared on the tiny holes on contact pad for connection. The extended gate was transferred to catheter and connected with transistor by using same method.

## Conflict of Interest

The authors declare no conflict of interest.

## Supporting information

Supplementary

Supplementary

Supplementary

Supplementary
