# Supplementary material for: Smart Surgical Catheter for C‐Reactive Protein Sensing Based on an Imperceptible Organic Transistor
Source: Adv Sci (Weinh). 2018 May 2;5(6):1701053. doi: 10.1002/advs.201701053 (PMC6010781; doi:10.1002/advs.201701053)
Supplement: Supplementary file 1 — Supplementary [file ADVS-5-1701053-s002.pdf]

Copyright WILEY-VCH Verlag GmbH & Co. KGaA, 69469 Weinheim, Germany, 2018.

ADVANCED SCIENCE
Open Access

Supporting Information

for *Adv. Sci.,* DOI: 10.1002/advs.201701053

Smart Surgical Catheter for C-Reactive Protein Sensing Based on an Imperceptible Organic Transistor

*Xudong Ji, Pengcheng Zhou, Ling Zhong, Aimin Xu, Anderson C. O. Tsang, and Paddy K. L. Chan**

Copyright WILEY-VCH Verlag GmbH & Co. KGaA, 69469 Weinheim, Germany, 2016.

Supporting Information

Smart Surgical Catheter for C-Reactive Protein Sensing Based on Imperceptible Organic Transistor

*Xudong Ji, Pengcheng Zhou, Ling Zhong, Aimin Xu, Anderson C. O. Tsang, and Paddy K. L. Chan**

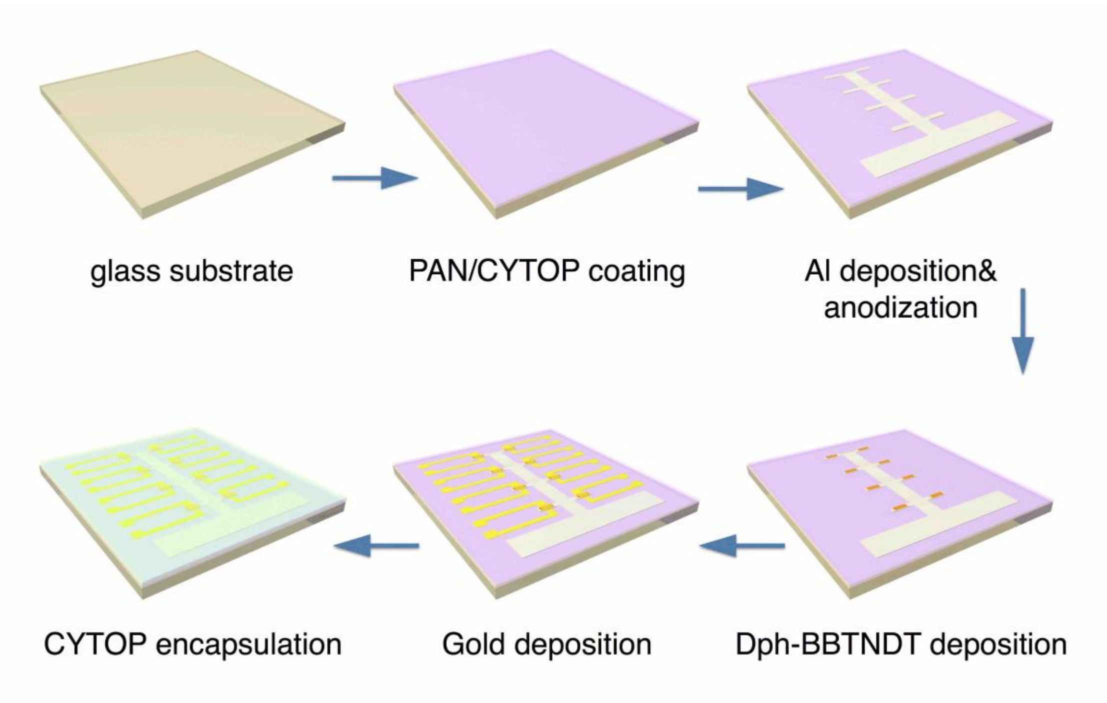

Figure S1. Fabrication process of ultra-thin OFET device.

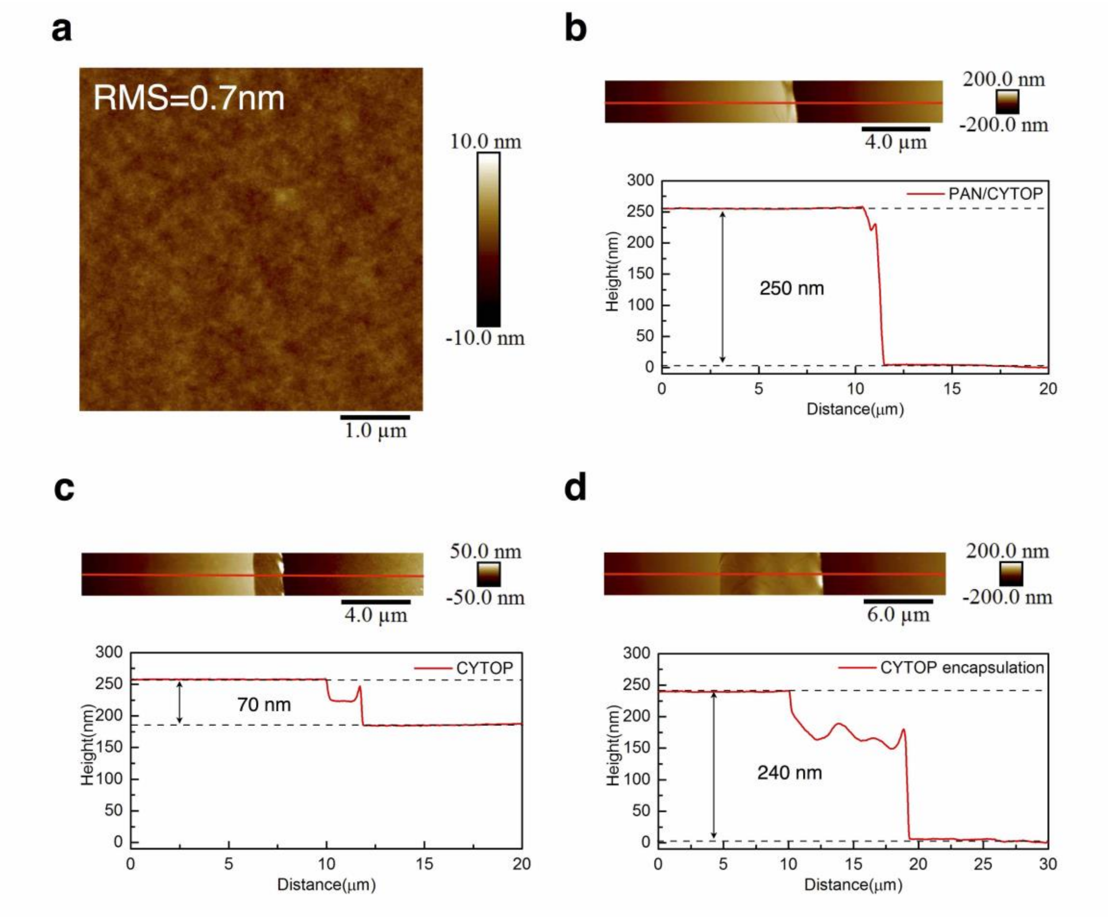

Figure S2. (a) AFM image of smooth surface of PAN/CYTOP hybrid substrate, RMS roughness is 0.7 nm; (b) AFM image along the edge of PAN/CYTOP double layer, thickness is 250 nm; (c) AFM image along the edge of a single CYTOP layer, thickness is 70 nm; (d) AFM image along the edge of top CYTOP encapsulation layer, thickness is 240 nm.

Characterization of anodized alumina dielectric

We formed high quality anodized growth alumina dielectric directly on the unconventional PAN/CYTOP hybrid substrate and compared its surface roughness with the one formed on glass substrate. The RMS roughness of alumina on PAN/CYTOP substrate is 1.6 nm which is just 0.5 nm higher than the alumina on glass substrate (Figure S3a, b). Metal-Insulator-Metal (MIM) structure was used to measure the area capacitance of alumina/ODPA SAM hybrid dielectric on PAN/CYTOP hybrid substrate. According to the capacitance frequency (C-F) measurement (Figure S3c), the area capacitance was evaluated to be around 370 nF cm^{-2} at 1 KHz which is high enough to ensure the low-voltage operation of transistor around 3 V.

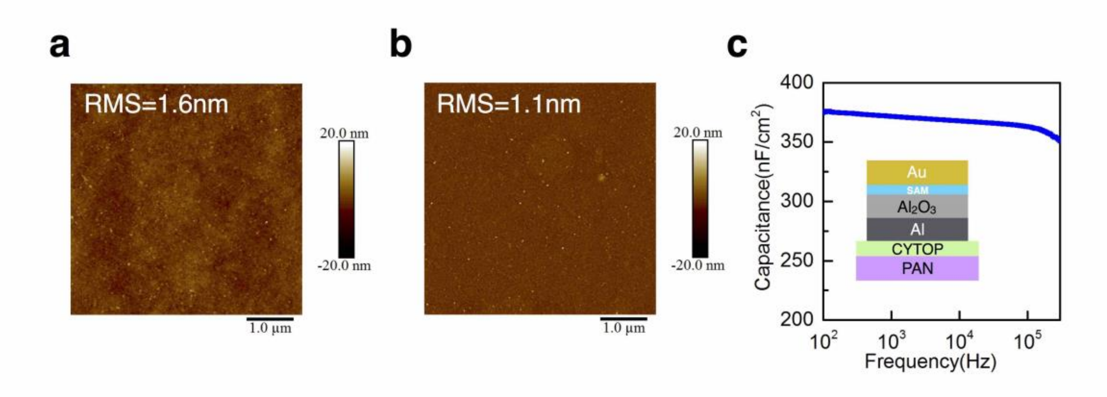

Figure S3. (a) AFM image of anodized alumina on PAN/CYTOP hybrid substrate, RMS roughness is 1.6 nm; (b) AFM image of anodized alumina on glass substrate, RMS roughness is 1.1 nm; (c) Capacitance-frequency measurement of alumina/ODPA SAM hybrid dielectric based on MIM structure on PAN/CYTOP substrate.

Thermal stability test of DPh-BBTNDT

We fabricated OFET devices based on OTS-treated Si/SiO_2 substrate with newly developed thermally stable organic semiconductor DPh-BBTNDT (Figure S4a). We evaluated the thermal stability of organic semiconductor by a 30 minutes' thermal annealing treatment with temperature ranging from 100 ℃ to 280 ℃ after device fabrication. The devices shown stably low off current around 30 pA at different temperature and only a slightly shift in threshold voltage (Figure S4b). By comparing the extracted saturation carrier mobility, we can conclude that when the temperature is below than 200 ℃, the carrier mobility degradation is negligible and can be maintained around 5 $cm^2\ V^{-1}\ s^{-1}$ (Figure S4c).

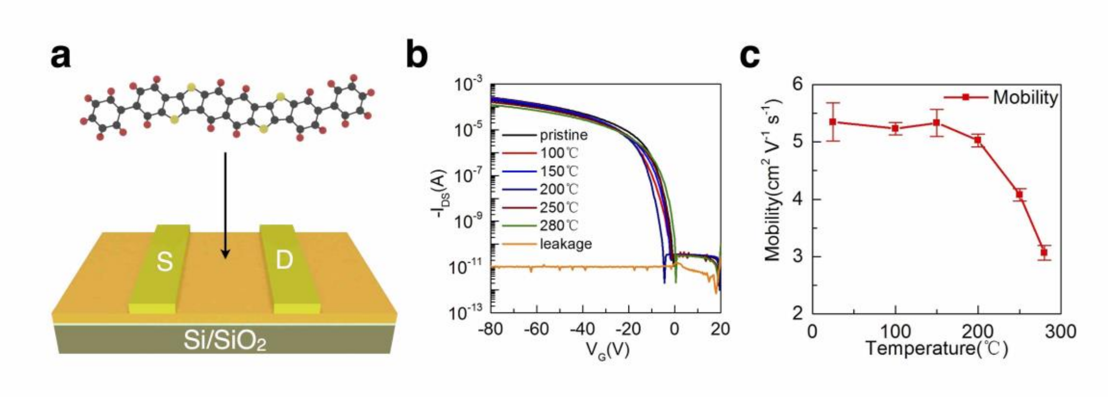

Figure S4. (a) Molecular structure of DPh-BBTNDT and the transistor based on OTS-treated Si/SiO_2 substrate; (b) Transfer curves of device after thermal annealing at different temperature; (c) Mobility change against annealing temperature; Scale bar is obtained from ten individual devices.

Device transfer mechanism and process

The ultra-thin device can be detached from the supporting frame by utilizing the hydrophobic/hydrophilic property of CYTOP/PAN hybrid substrate. Specifically, CYTOP is more hydrophobic with water contact angle 110°, while PAN is more hydrophilic with water contact angle 53° (Figure S5a). Thus, when immersing the device into water, the water goes into PAN/glass interface instead of PAN/CYTOP interface (Figure S5b). Due to this property, the device can be easily peeled off by simply immersing in water solution and then it will spread automatically on water surface. By using a targeted object to lift the floating device up, the transfer process can be executed.

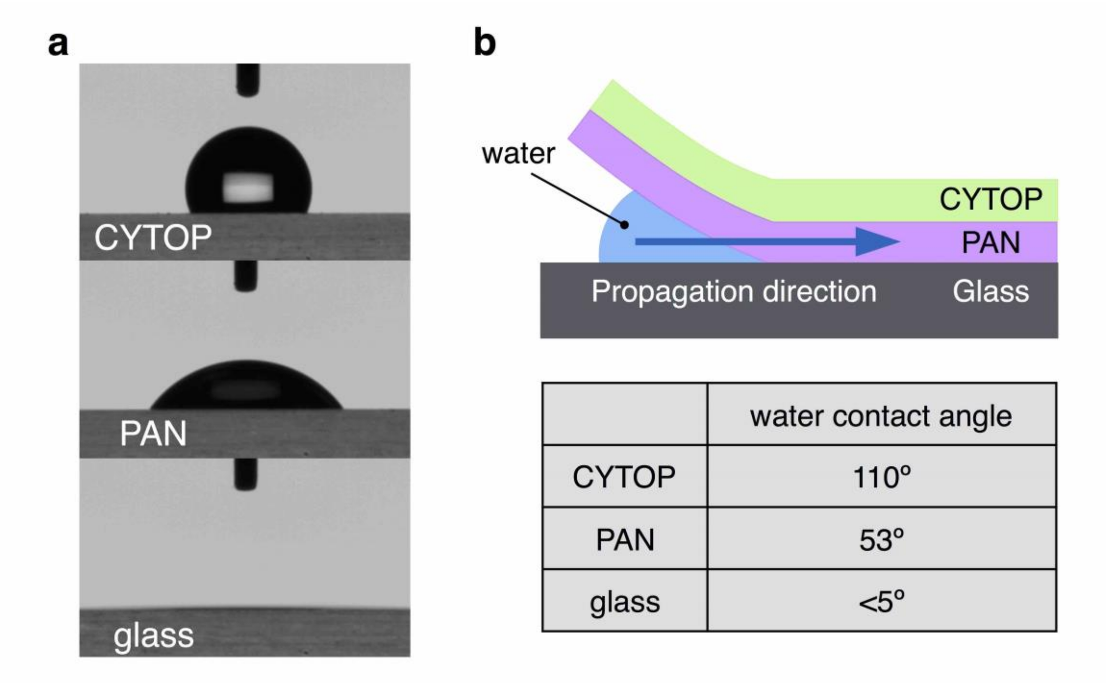

Figure S5. (a) Measurement of water contact angle on CYTOP, PAN and oxygen plasma-treated glass surface. The water contact angle is 110°, 53°, and less than 5°

respectively; (b) Schematic view of water floatation process for peeling off ultra-thin PAN/CYTOP substrate.

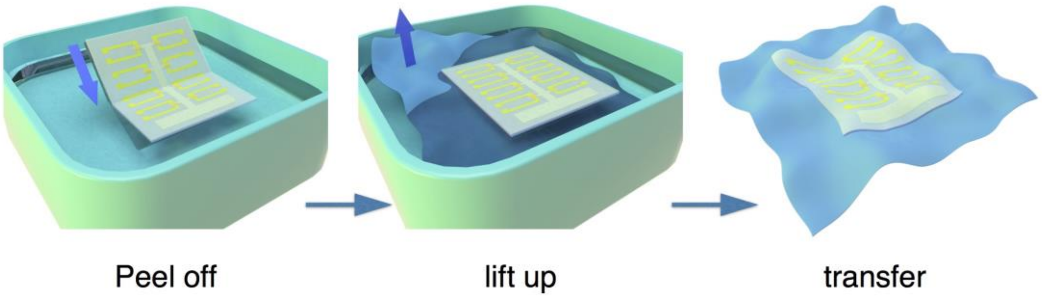

Figure S6. Transfer process of ultra-thin device.

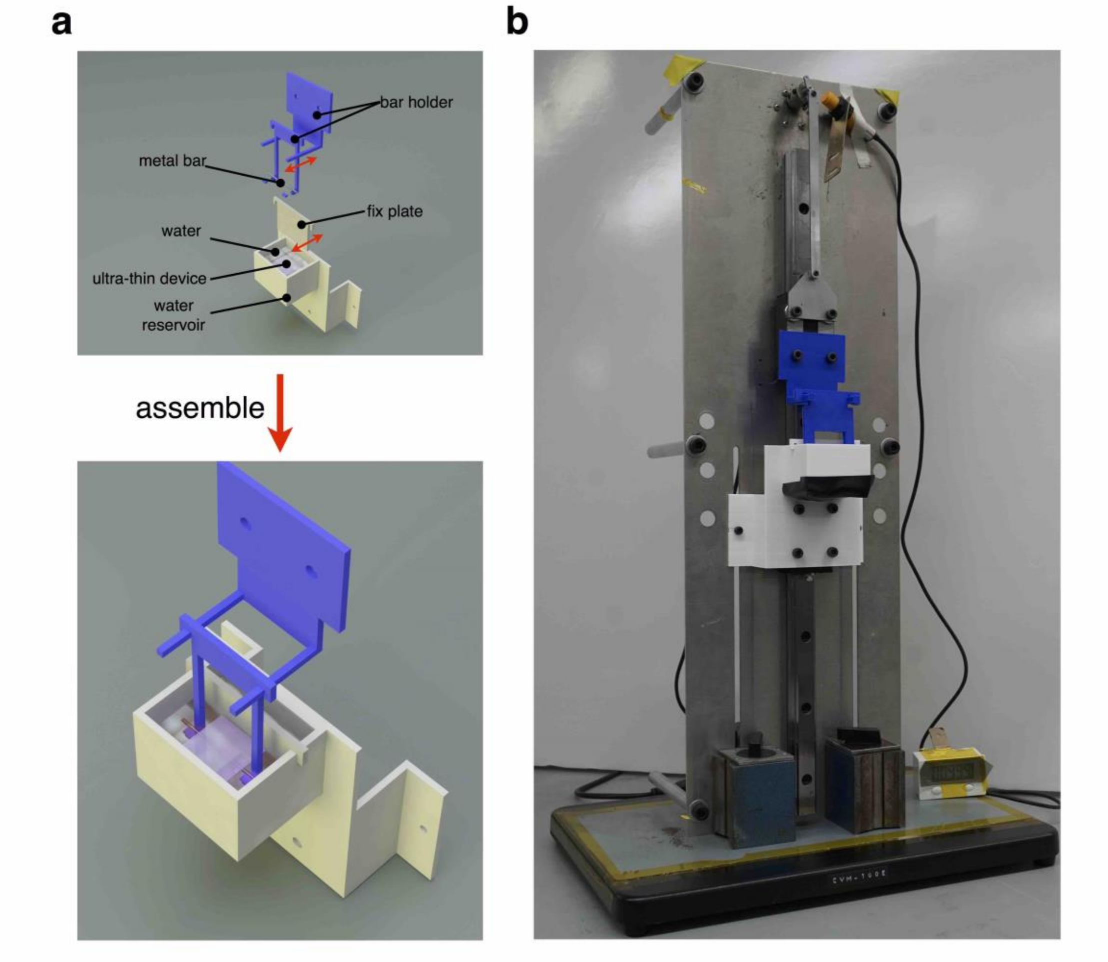

Figure S7. (a) Schematic view of different components in 3D printed bending machine; (b) Photograph of bending machine.

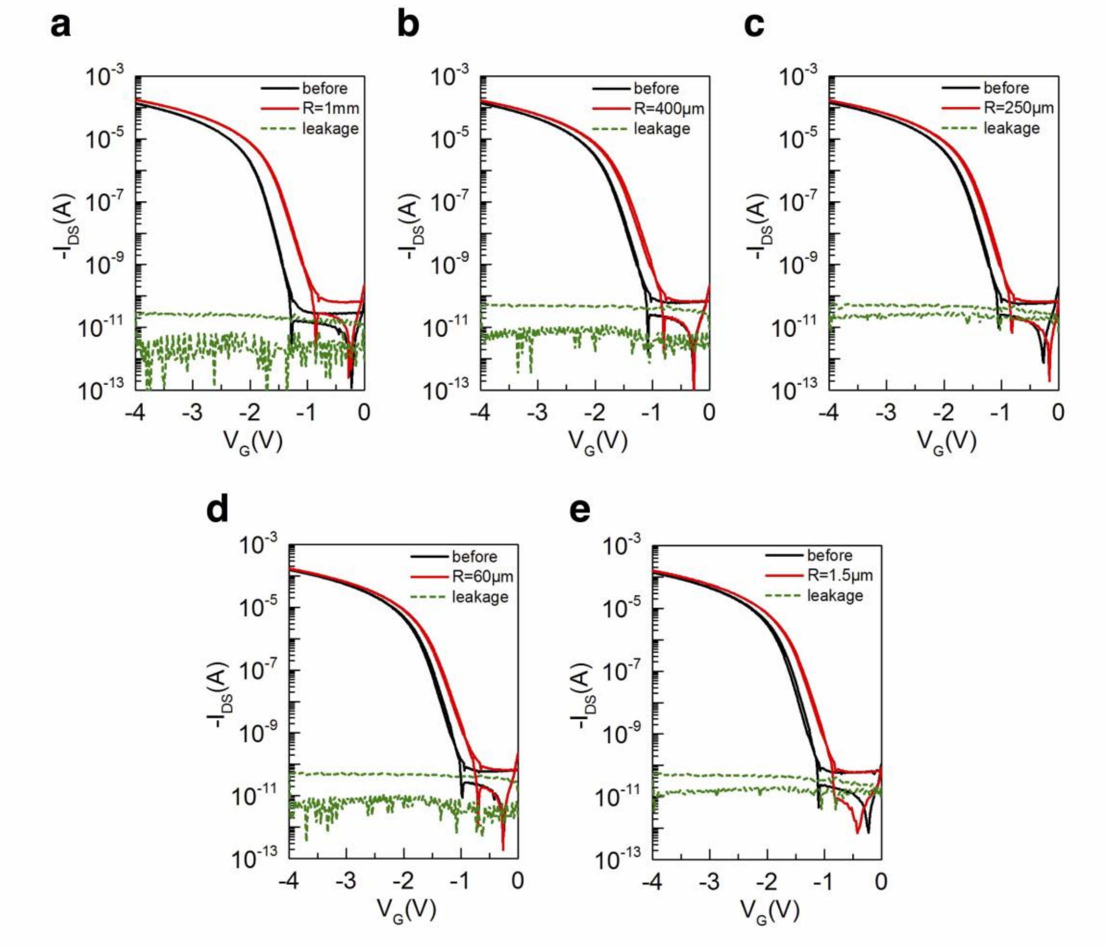

Figure S8. (a~e) Transfer curves before and after 5000 cycles bending test with different bending radius (1 mm, 400 μm, 250 μm, 60 μm and 1.5 μm respectively.) and leakage current after bending.

Strain analysis for ultra-thin transistor

We ignore the gold source drain electrode and only consider the strain in active channel at the strain model. The Young's modulus and thickness of each layer can be found in Table S1 and defined as h_i and Y_i $(i = 1, 2, 3, 4, 5, 6)$. When the total thickness of device is smaller than the bending radius, the bending induced strain at a position with distance r from the bottom of device can be defined as: $\varepsilon(r) = \frac{r - r_n}{R + r_n}$, where r_n is the distance between neutral mechanical plane (NMP) and the bottom surface of device. In pure bending without any external force applied, the circumferential stress through the thickness of device is in mechanical equilibrium, gives that $\int_0^h \sigma(r)dr = 0$. $h = \sum_{i=1}^{n} h_i$ is the total thickness of transistor and $\sigma(r)$ is the strain inducing stress which can be represented by Hooke's Law $\sigma(r) = Y_i \varepsilon(r)$. By solving this equitation, we can get the NMP position:

$$r_n = \frac{\sum_{i=0}^{n} Y_{i+1} h_{i+1} \left[\left(\sum_{j=0}^{i} h_j \right) + \frac{h_{i+1}}{2} \right]}{\sum_{i=0}^{n} Y_{i+1} h_{i+1}} \qquad \text{(S1)}$$

h_i is the thickness of layer i and $h_0 = 0$, we have assumed that the Poisson’s ratios of the layers are identical. By substituting the thickness and Young's modulus in this equation, we can get the position of NMP as: $r_n = 290\, nm$. The strain at the interface between dielectric layer and the semiconductor has a critical influence on transistor's performance and can be calculated as:

$$\varepsilon = \frac{h_1 + h_2 + h_3 + h_4 - r_n}{R + r_n} \qquad \text{(S2)}$$

So, the bending inducing strain in that interface can be written as: $\varepsilon = \frac{10}{R+290}$. Based on the above calculation, we can get a 0.56 % tensile strain when bending radius is 1.5 μm.

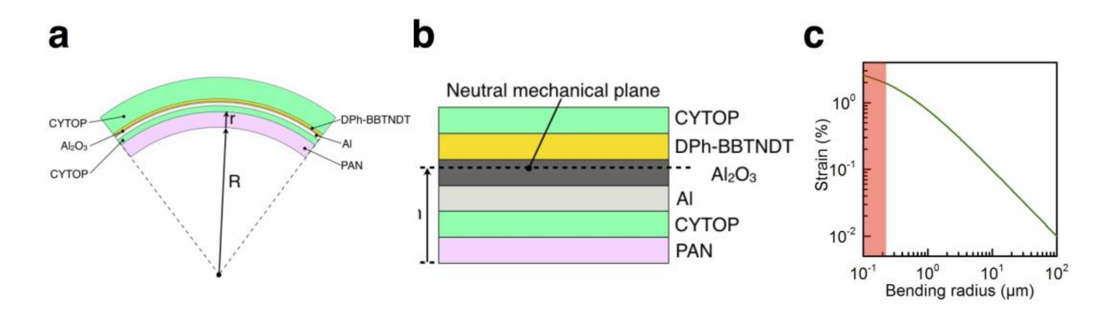

Figure S9. (a) Strain model of device with 6 layers (without gold layer), PAN, CYTOP, Al, Al_2O_3, DPh-BBTNDT and CYTOP (from bottom to top) under bending radius R; (b) Position of neutral mechanical plane (NMP); (c) Bending inducing strain against bending radius.

Extended gate characterization

To confirm the successfully immobilization of CRP antibody on extended gate, we first tested the contact angle of gold electrode before and after modified with 3-Mercaptopropionic acid (MPA) and functionalized with CRP antibody. We could clearly see that the contact angle of gold electrode was decreased from 58.8° to 20.6° after modified with MPA which is due to the hydrophilic property of MPA SAM. After functionalized with CRP antibody, the contact angle of gold electrode increased again to 47.5° due to the deposition of CRP antibody (Figure S10a). We also did the cyclic voltammetry (CV) test to validate the existence of CRP antibody (Figure S10b). The lower peak current density and broaden peak shape results from the decreased charge transfer rate in the gold electrode and electrolyte interface indicate an additional CRP antibody layer in the gold electrode surface.

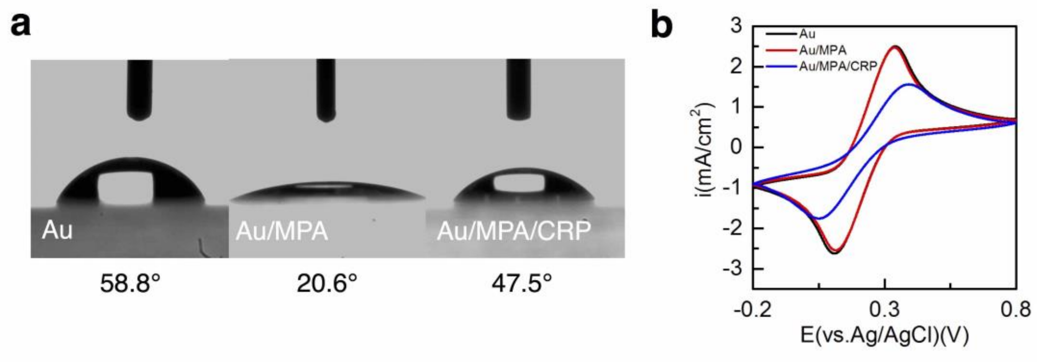

Figure S10. (a) Contact angle of extended gate before and after functionalized with MPA and CRP antibody; (b) CV test of extended gate before and after functionalized with MPA and CRP antibody.

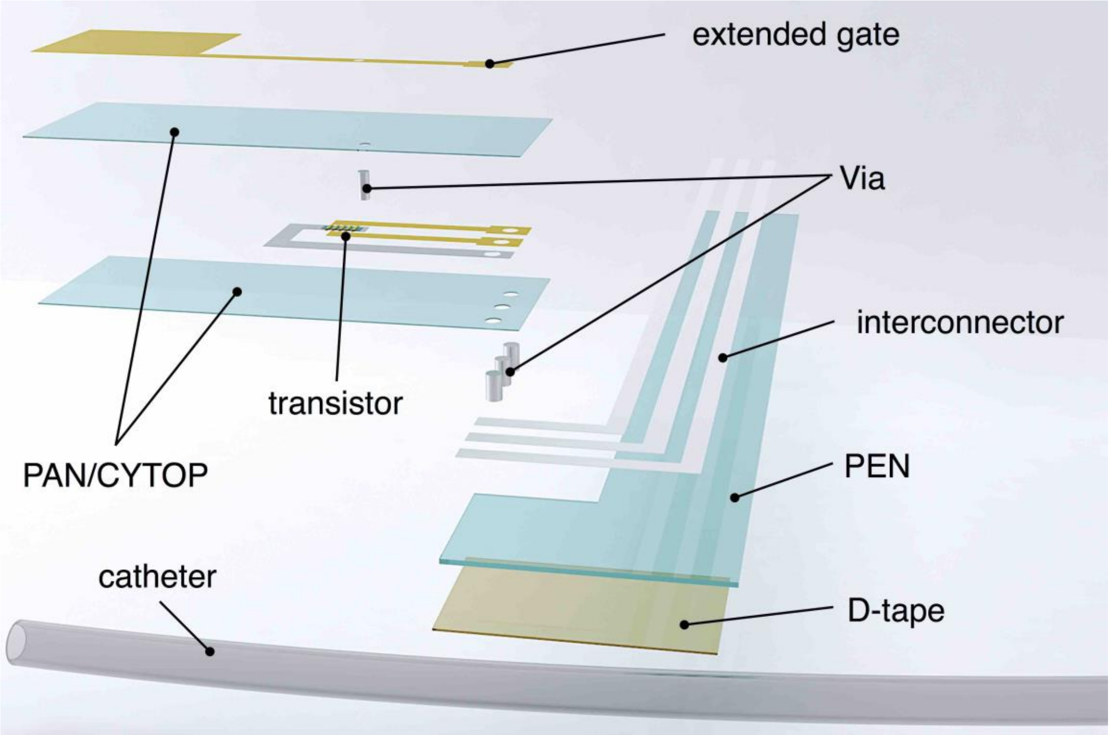

Figure S11. Exploded view of imperceptible organic transistor-based CRP sensor on ventricular catheter.

Table S1. Thickness and Young's modulus of each layer.

Layer index	Film	Thickness (nm)	Young's modulus (Gpa)
1	PAN	180	0.125
2	CYTOP	70	1.3
3	Al	40	69
4	Al_2O_3	10	300
5	DPh-BBTNDT	30	2.3
6	CYTOP	240	1.3

Table S2. Summary of the performance different biosensor for CRP detection.

	Structure	detection limit	reference
Electrical	Transistor - Carbon nanotube	10^{-4} μg/mL	1
	Impedance sensor - polycrystalline gold	1.94×10^{-2} μg/mL	2
	Transistor - Si	3 μg/mL	3
	Impedance sensor - carbon nanofiber	11 ng/mL	4
	Transistor - electrolyte gate with poly(3-hexylthiophene-2,5-diyl) (P3HT)	1 μg/mL	5
	Transistor - AlGaN/GaN	10 ng/mL	6
Mechanical	MEMS - Microcantilevel	1 μg/mL	7
	Piezoresistive microcantilevel	1 μg/mL	8
Optical	Surface plasmon resonator	0.01 μg/mL	9
Current work	Transistor - functionalized extended gate with Dph-BBTNDT	1 μg/mL	this work

Reference

[1] C. I. L. Justino, A. C. Freitas, J. P. Amaral, T. A. P. Rocha-Santos, S. Cardoso, A. C. Duarte, *Talanta* **2013**, *108*, 165.

[2] T.Bryan, X. Luo, P. R. Bueno, J. J. Davis, *Biosens. Bioelectron.* **2013**, *39*, 94.

[3] Y.-S. Sohn, Y. T. Kim, *Electron. Lett.* **2008**, *44*, 16.

[4] R. K. Gupta, A. Periyakaruppan, M. Meyyappan, J. E. Koehne, *Biosens. Bioelectron.* **2014**, *59*, 112.

[5] G. Palazzo, D. De Tullio, M. Magliulo, A. Mallardi, F. Intranuovo, M. Y. Mulla, P. Favia, I. Vikholm-Lundin, L. Torsi, *Adv. Mater.* **2015**, *27*, 911.

[6] H. H. Lee, M. Bae, S.-H Jo, J.-K. Shin, D. H. Son, C.-H. Won, H.-M. Jeong, J.-H. Lee, S.-W. Kang, *Sensors* **2015**, *15*, 18416

[7] C.-H. Chen, R.-Z. Hwang, L.-S. Huang, S.-M. Lin, H.-C. Chen, Y.-C. Yang, Y.-T. Lin, S.-A. Yu, Y.-S. Lin, *IEEE Trans. Biomed. Eng.* **2009**, *56*, 2

[8] Y.-K. Yen, Y.-C. Lai, W.-T. Hong, Y. Pheanpanitporn, C.-S. Chen, L.-S. Huang, *Sensors*, **2013**, *13*, 9653.

[9] W. Wang, Z. Mai, Y. Chen, J. Wang, L. Li, Q. Su, X. Li, X. Hong, *Sci. Rep.* **2017**, *7*, 16904.
